# Curcumin and *Boswellia* in Inflammatory Disorders: Immune Mechanisms, Bioavailability and Dose Comparability

**DOI:** 10.3390/nu18213511

**Published:** 2026-10-25

**Authors:** Sigrun Chrubasik-Hausmann, Julia Vlachojannis

**Affiliations:** Institute of Forensic Medicine, Faculty of Medicine and University Hospital, University of Freiburg, Albertstr. 9, 79104 Freiburg, Germany; jvlachojannis@gmail.com

**Keywords:** curcumin, boswellic acids, bioavailability, formulation technology, dose equivalence, pharmacokinetics, inflammation, immunomodulation

## Abstract

**Background/Objectives:** Curcumin and *Boswellia* preparations have been investigated for anti-inflammatory and immunomodulatory effects, but clinical translation is complicated by differences in composition, formulation and exposure. This narrative review examines mechanisms, pharmacokinetics and clinical evidence across immune-mediated, degeneration-associated and acute systemic inflammatory disorders, emphasizing dose comparability. **Methods:** Literature was identified through iterative PubMed searches and reference list screening, prioritizing recent systematic reviews and meta-analyses and selected primary clinical and pharmacokinetic studies. Experimental pathway effects, human biomarker changes and patient-relevant outcomes were evaluated separately. **Results:** Clinical findings suggest potential adjunctive symptomatic benefits, with a comparatively larger evidence base in knee osteoarthritis; findings in other indications remain heterogeneous, inconsistent or limited. Differences in active constituents, delivery technology and administered doses restrict comparisons across preparations. Higher systemic exposure does not by itself establish greater clinical efficacy, and neither pharmacokinetic bioequivalence nor clinically equivalent doses have generally been established across formulations. Local gastrointestinal effects and tissue distribution may also contribute to clinical outcomes. Interpretation is further constrained by small trials, overlapping evidence sources and reported industry relationships. Safety considerations include idiosyncratic liver injury associated with turmeric/curcumin products and potential drug interactions. **Conclusions:** Curcumin and *Boswellia* preparations show potential as adjunctive interventions, but formulation heterogeneity and limitations of the clinical evidence preclude general assumptions of interchangeability or clinical superiority based on increased bioavailability. Future research should prioritize formulation-specific human pharmacokinetics, dose-finding and adequately powered, independently replicated clinical trials, including direct comparisons where appropriate.

## 1. Introduction

Inflammatory and immune mechanisms contribute to the pathogenesis and progression of a wide range of acute and chronic disorders, including autoimmune diseases, osteoarthritis, dementia and infection [1]. Beyond the acute inflammatory response, SARS-CoV-2 infection has also been associated with an increased risk of subsequent autoimmune and other immune-mediated diseases [2,3]. These observations have stimulated interest in plant-derived bioactives that may influence inflammatory and immune pathways. Among the extensively investigated botanical constituents are curcuminoids derived from *Curcuma longa* and boswellic acids derived from *Boswellia* species. Both are also relevant to nutritional and nutraceutical research because they are incorporated into diverse dietary-supplement formulations.

In the clinical literature, “curcumin” may refer either to the individual compound or to curcuminoid mixtures and preparations whose content is expressed as curcumin equivalents. This review retains the terminology used in the original publications and distinguishes the preparation mass, total curcuminoid content and individual curcumin content wherever these are specified. 

Experimental studies have identified multiple molecular targets through which curcumin and boswellic acids may influence inflammatory signaling and innate and adaptive immune responses [4,5]. These findings provide a mechanistic rationale for clinical investigation, but do not themselves establish efficacy in humans. Likewise, improvements in pain, physical function or other clinical symptoms do not necessarily demonstrate a direct effect on immune function. Mechanistic findings, changes in inflammatory or immune biomarkers, and patient-relevant clinical outcomes therefore require separate consideration.

A central difficulty in interpreting the clinical literature is that preparations derived from the same botanical source are not necessarily pharmacologically comparable. Formulation technology, active constituent composition, administered dose and systemic exposure vary substantially. Similar nominal product doses may contain different amounts or forms of active constituents, while increased systemic exposure does not by itself establish greater clinical efficacy. These differences complicate the interpretation of pooled results, dose-based subgroup analyses and indirect comparisons between preparations.

The present narrative review is an integrated, critical appraisal of these determinants in relation to clinical evidence across three pathophysiologically distinct settings: immune-mediated inflammatory disorders, degeneration-associated inflammatory disorders, and acute systemic inflammatory disorders associated with immune dysregulation. Rather than treating curcumin or *Boswellia* preparations as interchangeable interventions, we examine how formulation characteristics and the doses actually administered affect the interpretation of clinical findings. Particular attention is given to divergent meta-analytic results, overlap of primary studies across reviews, discrepancies in reported doses, and the limitations of formulation-specific evidence.

This review addresses a cross-cutting question: to what extent can findings obtained with one preparation and dose inform the interpretation of findings obtained with another? Three concepts require distinction. Dose comparability refers here to the extent to which administered doses can be meaningfully compared in terms of active-constituent composition, formulation characteristics, dosing regimen and available exposure data. Pharmacokinetic bioequivalence means that two preparations containing the same active moiety produce sufficiently similar AUC and C_max_ values after administration of the same molar dose under comparable conditions, assessed against predefined acceptance limits [6]. AUC denotes the area under the concentration–time curve and C_max_ the maximum plasma concentration. For multicomponent botanical extracts, it must also be specified which constituents are measured and compared. Measuring a single constituent may not adequately characterize the preparation as a whole.

Clinical dose equivalence is used in this review as a working concept referring to doses of different preparations that provide equivalent clinical effects for a specified indication. Establishing such equivalence requires appropriately designed comparative trials with predefined, clinically justified equivalence margins; separate demonstrations of benefit over the placebo are insufficient. Because pharmacokinetic bioequivalence and clinical dose equivalence have generally not been established across curcumin and *Boswellia* preparations, this review focuses on dose comparability, while recognizing that higher systemic exposure does not necessarily mean a greater clinical benefit.

## 2. Methods

This narrative review critically examines formulation characteristics, bioavailability and dose comparability in relation to clinical evidence for curcumin and *Boswellia* preparations. The literature was identified through iterative searches in PubMed and examination of the reference lists of relevant publications during manuscript preparation in 2026, up to completion of the initial manuscript on 30 September 2026. The searches addressed mechanisms, formulation technology, pharmacokinetics and the selected clinical indications. No prospectively defined systematic search protocol was used, and exhaustive coverage of all primary studies was not intended. During revision, supplementary targeted PubMed searches and reference checks through 16 October 2026 addressed formulation-specific clinical sources, tissue exposure, safety and potential drug interactions.

Mechanistic aspects were based primarily on comprehensive reviews, supplemented by selected primary studies. Recent systematic reviews and meta-analyses formed the principal basis for the clinical evidence synthesis. Primary clinical and pharmacokinetic publications were examined selectively to clarify formulation composition, administered doses and findings not adequately addressed in the systematic reviews and meta-analyses. Earlier publications were considered where relevant. Selection of systematic reviews and meta-analyses was guided by relevance to the selected indications, recency and coverage of their searches, and availability of information on preparation characteristics, dosing and methodological limitations. “Recent” was a descriptive priority rather than a fixed publication-year eligibility criterion. Primary studies were selected to resolve these questions or to examine clinically relevant positive, negative or inconclusive findings; statistical significance was not an inclusion criterion.

For each preparation, the information considered included formulation technology, active-constituent composition, nominal daily dose, treatment duration and available pharmacokinetic findings. Preparations were grouped descriptively according to their formulation characteristics; these categories do not represent formal ratings of evidence certainty. Human pharmacokinetic findings were distinguished from preclinical findings and clinical outcomes.

Selected clinical studies were linked to the investigated preparations and indications. Nonrandomized studies and potentially overlapping reports were explicitly identified, and multi-ingredient botanical combinations were listed separately. Reports that might share participants were not assumed to represent independent trials. The selection was not intended as an exhaustive list, and the number of trials was not interpreted as a measure of evidence quality or efficacy. Limitations reported in the selected systematic reviews and meta-analyses, including heterogeneity, risk of bias and overlap of primary studies, were considered when interpreting their findings.

No protocol was registered, no new meta-analysis was performed, and no independent formal risk-of-bias assessment was undertaken. Generative AI (ChatGPT, OpenAI) was used for language editing, structural refinement and reference-formatting support. The authors reviewed and verified the scientific content, source selection, numerical data, interpretations and final wording.

## 3. Results

### 3.1. Immunomodulatory and Anti-Inflammatory Mechanisms of Curcumin and Boswellia

Evidence from in vitro and animal studies indicates that curcumin and boswellic acids can influence multiple, partly overlapping signaling pathways involved in immune regulation and inflammation [4,5,7]. These findings provide a rationale for clinical investigation, but do not establish that the same mechanisms are engaged in humans at the doses and systemic exposures achieved with the preparations used in clinical trials. In experimental systems, both compounds have been reported to interfere with nuclear factor kappa B (NF-κB)-dependent signaling, thereby reducing the expression of pro-inflammatory mediators, including cytokines such as TNF-α, IL-1β and IL-6, as well as chemokines. In addition, effects on inflammasome activation, particularly the NLRP3 inflammasome, as well as modulation of oxidative stress and redox-sensitive signaling pathways have been described [5,7,8].

Further mechanisms involve pathways regulating cytokine signaling and immune-cell activation. *Curcuma* has been extensively investigated for its effects on Janus kinase/signal transducer and activator of transcription (JAK/STAT) signaling, whereas boswellic acids have also been reported to influence STAT-dependent signaling, although this mechanism is less extensively characterized [4,5,7].

*Curcuma* and boswellic acids also modulate arachidonic-acid metabolism, a classical inflammatory pathway targeted pharmacologically by non-steroidal anti-inflammatory drugs and glucocorticoids. For *Boswellia*, acetyl-11-keto-β-boswellic acid has been extensively investigated for effects on 5-lipoxygenase-dependent leukotriene formation, while other boswellic acids also influence cyclooxygenase-related prostaglandin pathways; β-boswellic acid additionally inhibits cathepsin G, a neutrophil-derived serine protease involved in inflammatory tissue injury and immune regulation [5,9,10]. *Curcuma* likewise affects both cyclooxygenase- and lipoxygenase-dependent inflammatory signaling, although through partly different molecular mechanisms [5,7].

At the cellular level, experimental studies have reported effects of curcumin and boswellic acids on processes involved in innate and adaptive immune responses. For curcumin, experimental evidence includes modulation of macrophage activation and polarization as well as T-cell differentiation, particularly the Th17/Treg balance [11,12]. Effects on B-cell responses have also been described, although they are less extensively characterized [4]. For boswellic acids, the available evidence is more strongly centered on innate immune mechanisms, leukocyte activity and inflammatory mediator production [5].

Taken together, these experimental findings identify potential anti-inflammatory and immunomodulatory mechanisms, but their clinical relevance must be evaluated separately for each preparation and indication. Translation depends on the active constituent composition, the concentrations achieved at the relevant site of action, and the treatment duration. A pathway affected in an experimental model cannot be assumed to mediate a clinical response without supporting human evidence.

Three levels of evidence should therefore be distinguished: experimental effects on molecular or cellular pathways; changes in inflammatory or immune biomarkers in humans; and improvements in patient-relevant clinical outcomes. Changes in circulating cytokines may support biological activity, but do not by themselves establish beneficial modulation of immune function or identify the mechanism responsible for a clinical effect. Conversely, improvements in symptoms do not demonstrate direct effects on immune regulation. Mechanistic plausibility should thus inform the interpretation of clinical findings without substituting for evidence of efficacy.

### 3.2. From Mechanism to Exposure: The Bioavailability Problem 

Due to poor aqueous solubility, variable intestinal absorption and extensive presystemic metabolism, both curcuminoids and boswellic acids exhibit limited oral bioavailability and highly variable systemic exposure. Numerous formulation strategies have therefore been developed to improve curcumin bioavailability, including phospholipid complexes, submicron and micellar dispersions, solid-lipid systems, natural carrier matrices and cyclodextrin complexes. Clinically investigated preparations based on these technologies include Meriva^®^, Theracurmin^®^, curcuRouge^®^, Longvida^®^, NovaSOL^®^, BCM-95^®^/BioCurcumin^®^, Cureit™, CurQfen^®^, Cavacurmin^®^/CW8 and CuroWhite™ (Table 1). CuroWhite™ differs additionally in containing predominantly hydrogenated curcuminoids rather than native curcumin. Comparative human pharmacokinetic studies demonstrate marked formulation-dependent differences in systemic exposure. In a randomized crossover study comparing eight curcumin formulations under standardized conditions, Flory et al. [13] found that micellar curcumin and a γ-cyclodextrin complex produced markedly higher systemic exposure than native curcumin at an equivalent curcumin dose, whereas the other formulations did not show a statistically significant increase in AUC versus native curcumin in that comparison. Only conjugated curcumin was detected; free curcumin was not detected.

For *Boswellia*, formulation strategies developed to enhance systemic exposure include phospholipid complexes, micellar preparations, self-emulsifying systems, lipid-based particles, polymeric carriers and cyclodextrin complexes [14]. Clinically investigated preparations include the phospholipid complex Casperome^®^, the micellar boswellia-Loges^®^ formulation, AKBA-enriched extracts such as 5-Loxin^®^ and Aflapin^®^, and the standardized *Boswellia serrata* extract K-Vie™ (Table 1). Although no single comprehensive comparison across multiple *Boswellia* delivery technologies is available, individual comparative studies demonstrate marked formulation-dependent differences in systemic exposure. Lecithin-complexed *Boswellia* extract increased the absorption of several boswellic acids compared with non-formulated extract in a randomized crossover study in healthy volunteers [15], while a micellar *Boswellia* preparation substantially increased Cmax and AUC and shortened Tmax for multiple boswellic and lupeolic acids compared with a native extract [16]. Interpretation is further complicated by the markedly different pharmacokinetic behavior of individual boswellic acids. *Boswellia* extracts differ not only in total boswellic-acid content but also in the relative amounts of β-boswellic acid and its acetylated derivatives, KBA and AKBA. Human pharmacokinetic studies indicate that β-boswellic acid reaches substantially higher circulating concentrations than AKBA [15,16]. Because individual boswellic acids differ markedly in systemic exposure and molecular targets, the nominal dose of a *Boswellia* extract does not reflect its pharmacological profile. Consequently, equal nominal doses of curcumin or *Boswellia* extract cannot be assumed to represent equivalent pharmacological doses across formulations (Table 2).

**Table 1:** *Curcuma* and *Boswellia* preparations: composition, selected doses, and human versus preclinical pharmacokinetic evidence. AKBA, acetyl-11-keto-β-boswellic acid; AUC, area under the concentration–time curve; Bs, *Boswellia serrata*; C_max_, maximum plasma concentration; PK, pharmacokinetics; UC, ulcerative colitis.

Preparation	Formulation and Composition	Clinical Daily Dose Examples	Dose in Cited Human PK Study	PK Evidence and Limitations	Refs.
Meriva^®^	Curcuminoid–phosphatidylcholine complex (Phytosome^®^) (Bioenhanced)	Not specified here; see indication-specific studies.	207 mg curcumin, single dose; *n* = 12 healthy adults.	Human randomized crossover: no statistically significant AUC increase versus native curcumin in this comparison. This does not exclude different findings under other study conditions. No free curcumin was detected; findings concern conjugated curcumin measured after sample processing.	[13]
Longvida^®^	Solid-lipid curcumin particle (SLCP™) (Bioenhanced)	Not specified here; see indication-specific studies.	207 mg curcumin, single dose; *n* = 12 healthy adults.	Human randomized crossover: no statistically significant AUC increase versus native curcumin in this comparison. This does not exclude different findings under other study conditions. No free curcumin was detected; findings concern conjugated curcumin measured after sample processing.	[13]
Theracurmin^®^	Submicron colloidal curcumin dispersion (Bioenhanced)	Not specified here; see indication-specific studies.	207 mg curcumin, single dose; *n* = 12 healthy adults.	Human randomized crossover: no statistically significant AUC increase versus native curcumin in this comparison. This does not exclude different findings under other study conditions. No free curcumin was detected; findings concern conjugated curcumin measured after sample processing.	[13]
curcuRouge^®^	Amorphous solid dispersion of curcumin (Bioenhanced)	Not specified here; see indication-specific studies.	90 mg curcumin, single dose.	Human crossover versus Theracurmin: reported AUC 3.4-fold and C_max_ 5.4-fold at equal curcumin dose. Clinical superiority not established.	[17]
NovaSOL^®^	Micellar curcumin (Bioenhanced)	Not specified here; see indication-specific studies.	207 mg curcumin, single dose; *n* = 12 healthy adults.	Human randomized crossover: conjugated curcumin AUC increased 57-fold versus native curcumin. No free curcumin was detected; findings concern conjugated curcumin measured after sample processing.	[13]
Cavacurmin^®^/CW8	*Curcuma*-γ-cyclodextrin complex (Bioenhanced)	Not specified here; see indication-specific studies.	207 mg curcumin, single dose; *n* = 12 healthy adults.	Human randomized crossover: conjugated curcumin AUC increased 30-fold versus native curcumin. No free curcumin was detected; findings concern conjugated curcumin measured after sample processing.	[13]
CuroWhite™	Hydrogenated curcuminoids in β-cyclodextrin (Chemically modified)	250 or 500 mg preparation/day, corresponding to approximately 63.3 or 126.7 mg hydrogenated curcuminoids/day (calculated from the reported composition: 20.8% THC + 3.7% HHC + 0.83% OHC = 25.33%) [18].	—	Ref. [18] reports clinical efficacy, not comparative human PK. Hydrogenated curcuminoids are chemically distinct from native curcumin; no PK equivalence inferred.	[18]
CurQfen^®^	Curcuminoids in fenugreek galactomannan matrix (Bioenhanced)	Not specified here; see indication-specific studies.	Food-matrix protocol; exact dose not reproduced here.	Ref. [19] includes a human bioavailability component and preclinical safety experiments. Food-matrix results should not be presented as PK evidence for a 400 mg clinical capsule regimen. Exact dose and matrix must be retained when quoting quantitative comparisons.	[19]
BCM-95^®^/BioCurcumin^®^	Curcuminoids + turmeric essential oil (Bioenhanced)	Not specified here; see indication-specific studies.	207 mg curcumin, single dose; *n* = 12 healthy adults.	Human randomized crossover: no statistically significant AUC increase versus native curcumin in this comparison. This does not exclude different findings under other study conditions. No free curcumin was detected; findings concern conjugated curcumin measured after sample processing.	[13]
Cureit™	Curcuminoids in proprietary turmeric matrix (Matrix-enhanced)	Not specified here; see indication-specific studies.	500 mg preparation, single dose; *n* = 15 healthy participants.	Human plasma concentration–time characterization. This study alone does not establish increased exposure versus a comparator.	[20]
Casperome^®^/Boswellia Phytosome^®^	Standardized Bs extract + phospholipids (Phytosome^®^) (Bioenhanced)	250 mg/day in the observational UC study [21].	500 mg preparation (2 × 250 mg), single dose; *n* = 12.	Human randomized crossover versus non-formulated extract: increased absorption of several boswellic acids. The PK dose is distinct from the observational clinical dose.	[15,21]
boswellia-Loges^®^	Micellar Bs formulation (Bioenhanced)	Not specified here; see indication-specific studies.	800 mg preparation, single dose; *n* = 20 healthy volunteers.	Human comparative PK/PD study: increased exposure to several boswellic and lupeolic acids. Exposure differences do not establish clinical efficacy.	[16]
Aflapin^®^/AprèsFlex^®^	AKBA-enriched Bs extract + non-volatile *Boswellia* oil (Compositionally optimized /bioenhanced)	100 mg preparation/day; standardized to ≥20% AKBA.	—	Rat PK evidence for increased AKBA availability. No comparative human PK study established by the cited sources.	[22,23,24]
5-Loxin^®^	Bs extract selectively enriched in AKBA (Standardized/compositionally enriched)	100 or 250 mg preparation/day; 30% AKBA (30 or 75 mg).	—	Ref. [25] is a clinical dose-ranging efficacy study, not a human PK study.	[25]
K-Vie™	*Boswellia serrata* extract; standardized to 40% boswellic acids (Standardized)	1200 mg preparation/day; standardized to 40% boswellic acids.	—	Ref. [26] is a clinical efficacy study; comparative human PK is not established by this source.	[26]

**Table 2:** Formulation-related determinants of pharmacological dose comparability for curcumin and *Boswellia* preparations. Formulation-specific pharmacokinetic characteristics and representative source references are summarized in Table 1.

Determinant	*Curcuma*	*Boswellia*	Implication for Dose Comparability
Formulation technology	Bioavailability-enhancing technologies differ substantially between preparations.	Phospholipid, micellar and compositionally optimized preparations differ in delivery technology.	Nominal doses are not directly comparable.
Active constituent composition	Preparations differ in curcuminoid content and composition; CuroWhite™ contains predominantly hydrogenated curcuminoids.	*Boswellia* extracts differ in total boswellic-acid content and in the relative amounts of β-boswellic acid and its acetylated derivatives, KBA and AKBA [5,15,16].	Equal extract or marker-compound doses do not imply equivalent pharmacological exposure.
Comparative human pharmacokinetics	Available for selected studies only.	Available for selected studies only.	PK differences characterize exposure, not clinical dose equivalence.
Clinical dose equivalence and dose ranging	Some dose-ranging trials are available; optimal doses and clinical dose equivalence across preparations remain insufficiently established.	Some dose-ranging trials are available; optimal doses and clinical dose equivalence across preparations remain insufficiently established.	Cross-formulation differences cannot be interpreted as dose–response relationships.
Exposure–effect relationship	Higher systemic exposure has not been shown to predict greater clinical efficacy across formulations.	Higher exposure need not translate into greater biological activity, and the same nominal dose may yield different clinical responses.	Exposure, composition and clinical effect must be interpreted together.

Abbreviations: AKBA, acetyl-11-keto-β-boswellic acid; KBA, 11-keto-β-boswellic acid; PK, pharmacokinetics.

**Figure 1:**
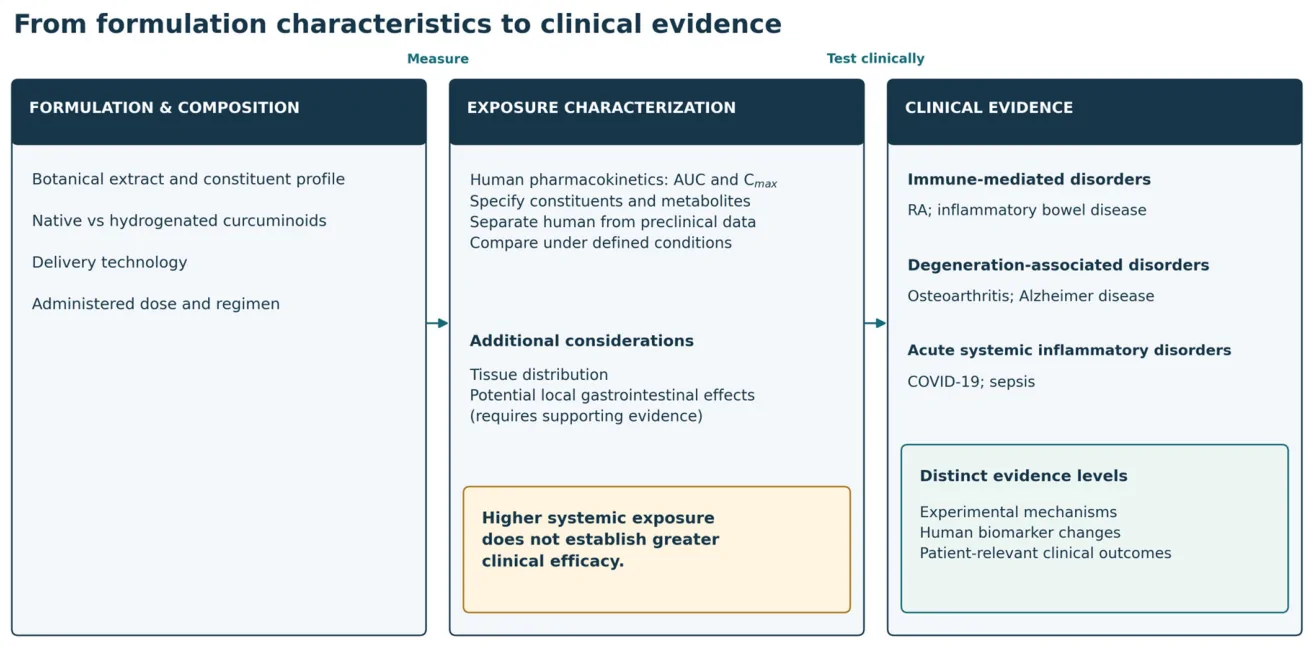
Framework linking formulation characteristics, exposure assessment and clinical evaluation. Arrows indicate stages of evaluation, not proof that higher exposure causes clinical benefit. Potential local gastrointestinal effects and tissue distribution require supporting evidence. Experimental mechanisms, human biomarkers and clinical outcomes represent distinct evidence levels. AUC, area under the concentration–time curve; C_max_, maximum plasma concentration; RA, rheumatoid arthritis.

Figure 1 summarizes the framework linking formulation characteristics, exposure assessment and clinical evaluation across the three indication groups, emphasizing that increased systemic exposure alone does not establish greater clinical efficacy.

### 3.3. Clinical Evidence Across Selected Inflammatory and Immune-Related Disorders

The following sections examine formulation-specific clinical evidence for curcumin and *Boswellia* across three pathophysiologically distinct settings. The term “degeneration-induced inflammatory disorders” is used here pragmatically to describe conditions in which degenerative tissue changes are accompanied by and can promote sustained inflammatory responses; this does not imply a strictly unidirectional relationship between degeneration and inflammation. Table 3 provides an overview of the selected clinical studies. The following sections discuss the findings and limitations for each indication.

### 3.4. Immune-Mediated Inflammatory Disorders

#### 3.4.1. Rheumatoid Arthritis (RA)

Recent meta-analyses have yielded divergent results regarding the efficacy of curcumin in RA. Fan et al. [27] reported significant improvements in disease activity, inflammatory markers and clinical symptoms, although the certainty of evidence was predominantly very low. In contrast, Liu et al. [77] found no significant pooled effects on DAS28, ESR or CRP and reported substantial between-study heterogeneity. The two meta-analyses were based on partly different sets of trials and included pharmacologically distinct curcumin preparations. Fan et al. included Cureit™, CuroWhite™ and nanomicellar formulations, whereas Liu et al. additionally included the earlier trial by Chandran and Goel [31] but did not include CuroWhite™ and the more recent study by Khamar et al. [29]. Importantly, nominal doses reported in the meta-analyses were not always consistent with the original publications. For example, Javadi et al. [30] administered 40 mg of nanomicellar curcumin three times daily (120 mg/day), whereas both meta-analyses listed a dose of 40 mg/day. Similarly, the 250 and 500 mg doses in the Amalraj trial [28] were administered twice daily. These discrepancies further complicate comparisons across trials using different formulations and dosing regimens. The Cureit trial included only 36 participants (12 per group) [28], and the CuroWhite trial included 24 (eight per group) [18]. These small samples limit precision and the reliability of effect-size estimates and require confirmation in larger trials.

Clinical evidence for *Boswellia* in RA is limited and derives mainly from older studies using the standardized *Boswellia serrata* extract H15. Early controlled trials suggested potential improvements in joint symptoms and inflammatory parameters, but sample sizes were small and methodological quality was limited [9]. A later reassessment of the available data did not confirm a robust superiority over the placebo, and current rheumatological recommendations consider the evidence insufficient for firm conclusions regarding efficacy in inflammatory RA [78].

#### 3.4.2. Inflammatory Bowel Disease (IBD)

Recent meta-analyses support a potential adjunctive role of curcumin in ulcerative colitis. Peng et al. [79] included eight randomized controlled trials with 482 patients and found significant improvements in clinical remission and clinical response, whereas the increase in endoscopic remission did not reach statistical significance. Heterogeneity was substantial for several outcomes. A broader meta-analysis by Mohseni et al. [32], including 13 placebo-controlled IBD study reports, likewise found significant benefits for clinical remission and response in ulcerative colitis. For Crohn’s disease, however, the pooled analysis of only two trials—one using the bioavailability-enhanced formulation Theracurmin^®^—found no significant advantage over the placebo for clinical or endoscopic remission. Table 3 identifies selected clinical reports by preparation and indication. It excludes the *Curcuma*–QingDai combination and the rectal enema study from the oral-curcumin entries and flags the possible overlap between Banerjee 2017 and 2021 [33,34]. The reported meta-analytic findings refer to the original authors’ analyses and were not recalculated for this selection.

Clinical evidence for *Boswellia serrata* in IBD is limited and heterogeneous [9]. In an early randomized trial in patients with active Crohn’s disease, a *Boswellia serrata* extract (H15, 3.6 g/day) was associated with a reduction in the Crohn’s Disease Activity Index comparable to that observed with mesalazine [38]. In contrast, a subsequent randomized placebo-controlled trial in patients with Crohn’s disease in remission found no benefit of *Boswellia serrata* extract (Boswelan, 2.4 g/day) for the prevention of relapse over 52 weeks [39].

Interpretation is further complicated by substantial differences in the *Boswellia* preparations investigated. Earlier trials used conventional *Boswellia serrata* preparations at gram doses, whereas the only clinical study in ulcerative colitis using a clearly bioavailability-enhanced formulation evaluated the lecithin-based *Boswellia* preparation Casperome^®^ at 250 mg/day. However, this was an open-label observational study without randomization or placebo control, which substantially limits conclusions regarding efficacy [21]. Overall, the available findings suggest potential clinical activity, but the evidence remains insufficient to establish formulation-specific efficacy or an optimal dose.

### 3.5. Degeneration-Induced Inflammatory Disorders

#### 3.5.1. Osteoarthritis (OA)

Clinical evidence for curcumin in knee OA is comparatively extensive. In a recent umbrella review of ten systematic reviews and meta-analyses, Shi et al. [80] found consistent evidence of abenefit for pain and joint function, with effects in several analyses comparable to NSAIDs and a tendency towards better tolerability. However, only three of the included reviews were rated as being of high methodological quality, and substantial clinical and methodological heterogeneity was present. Shi et al. reported 38 unique primary RCTs, occurring 133 times across the included reviews, with a corrected covered area of 27.8%, indicating substantial overlap. These counts are reported by the umbrella-review authors and are not a formulation-specific census performed for the present narrative review. Table 3 identifies selected clinical studies and the preparations investigated. Thus, the apparent consistency across reviews should not be interpreted as independent evidence of efficacy. The investigated interventions were pharmacologically heterogeneous and included conventional curcuminoids as well as multiple bioavailability-enhanced preparations ( Table 1; Table 3). Some trials investigated more than one dose of the same preparation, but optimal formulation-specific doses and clinical dose equivalence across preparations remain insufficiently established. Beyond symptomatic outcomes, Hsueh et al. [81] evaluated serum inflammatory biomarkers in 21 randomized controlled trials and found significant reductions in CRP and a borderline significant reduction in TNF-α, whereas ESR, IL-1β, IL-6 and PGE_2_ were not significantly affected. In total, 12 of the 21 included trials used techniques intended to enhance curcumin bioavailability.

The distinction between symptomatic and inflammatory outcomes is illustrated by the randomized trial of Wang et al. In 70 participants with symptomatic knee osteoarthritis and effusion-synovitis, *Curcuma longa* extract improved pain over 12 weeks but did not significantly affect the MRI-assessed effusion-synovitis volume or cartilage composition. Symptomatic improvement therefore did not coincide with an improvement in these imaging outcomes [40].

Clinical evidence for *Boswellia serrata* in knee OA is also supportive, although the number of trials is smaller than for curcumin. In a meta-analysis of seven randomized controlled trials including 545 patients, Yu et al. [82] reported significant improvements in VAS pain, WOMAC pain, stiffness and physical function compared with control interventions. The *Boswellia* preparations investigated included conventional extracts as well as AKBA-enriched and bioavailability-enhanced formulations ( Table 1; Table 3). Particularly illustrative are 5-Loxin^®^ and Aflapin^®^. 5-Loxin^®^ is a *Boswellia serrata* extract enriched to 30% AKBA and was investigated at 100 and 250 mg/day in a randomized placebo-controlled trial [25]. Aflapin^®^ combines an AKBA-enriched *Boswellia* extract with a non-volatile *Boswellia* oil fraction. In a direct randomized comparison, both Aflapin^®^ and 5-Loxin^®^ were administered at the same nominal dose of 100 mg/day; both significantly improved pain and physical function, with some outcomes favoring Aflapin^®^ in this small trial [22]. A further randomized placebo-controlled trial confirmed clinical improvements with Aflapin^®^ at 100 mg/day [23]. Preclinical pharmacokinetic studies in rats reported greater systemic AKBA availability from Aflapin^®^ than from 5-Loxin^®^ [24], illustrating that the nominal extract dose and AKBA content alone do not necessarily predict pharmacological exposure or clinical efficacy.

An earlier systematic review by Bannuru et al. included 14 randomized trials, of which 11 involving 1009 participants contributed to meta-analyses. Both curcuminoid and *Boswellia* preparations showed benefits over the placebo for pain and function, but the overall study quality was low. Industry sponsorship or direct industry involvement was reported in 6 of the 11 quantitatively analyzed trials. These findings support a potential symptomatic benefit while emphasizing the need for more robust evidence; they do not establish comparative superiority between the two botanicals [83].

Network meta-analyses have yielded favorable rankings for *Boswellia*-containing interventions, but these findings require formulation-level interpretation. Zhang et al. [84] included 39 randomized trials involving 4599 patients across seven nutritional intervention categories; this total is not the number of curcumin and *Boswellia* trials alone. Their eight *Boswellia*-labeled study entries included the multi-ingredient formulations Curamin and LI73014F2. The latter contains *Terminalia chebula*, *Curcuma longa* and *Boswellia serrata* extracts. Thus, this category does not exclusively represent *Boswellia*-only preparations, and its ranking cannot establish the comparative efficacy of *Boswellia* alone. Inprasit et al. [85] included 20 randomized trials involving 1633 participants and separately grouped *Curcuma*, *Boswellia* and combination interventions, including modified formulations. Modified *Boswellia* formulations showed favorable WOMAC outcomes, while modified *Curcuma* formulations reduced VAS pain compared with the placebo. However, the formulation composition, nominal dose, comparator and treatment duration differed between trials, direct comparisons were limited, and clinical dose equivalence has not been established. The findings therefore do not establish intrinsic superiority of either botanical or clinical superiority attributable to enhanced bioavailability.

Overall, both curcumin and *Boswellia* show evidence of a clinically relevant benefit in knee OA, but formulation heterogeneity, methodological limitations and uncertainty regarding dose equivalence preclude firm conclusions regarding the superiority of individual preparations or of one botanical over the other.

Small individual studies also constrain the OA evidence. For example, the Longvida pilot study recruited 50 participants, of whom 42 completed treatment [51]. Such studies can provide preliminary signals but have limited precision for treatment effects and limited ability to characterize uncommon adverse events. Their results should not be interpreted as establishing clinical superiority between formulations.

The trials of 5-Loxin^®^ and Aflapin^®^ cited here received industry funding or support, and some included investigators with company affiliations or consultancy relationships [22,23,25]. These relationships do not invalidate the findings, but should be considered alongside the sample size, treatment duration and methodological limitations. Additional replication by independent research groups would strengthen our confidence in formulation-specific effects. Industry relationships should be assessed from the primary publications rather than inferred to be absent from a review-table entry labeled “No”. The newly identified trials and formulations likewise require consideration of their reported funding and investigator affiliations.

##### Additional Evidence in Spinal Disorders

Additional evidence for *Boswellia* preparations comes from a trial in patients with spondylitis/spondylosis. This heterogeneous population is distinct from rheumatoid arthritis and peripheral osteoarthritis; its findings should therefore be considered separately from the evidence for these conditions. A randomized, double-blind, placebo-controlled three-arm trial investigated a bioavailability-enhanced full-spectrum *Boswellia serrata* extract (F-BSE) and a co-delivery formulation of the same extract with curcumin (C-BSE) in 105 participants with moderate spondylitis/spondylosis. Although an a priori power calculation providing 95% statistical power was reported, the calculation was not sufficiently reproducible from the information provided. Participants received 400 mg/day for 28 days. Both active treatments significantly improved the Bath Ankylosing Spondylitis Disease Activity Index (BASDAI) and Neck Disability Index (NDI) and reduced NLRP3 and IL-1β levels compared with the placebo. The combination formulation containing curcumin produced greater improvements than *Boswellia* alone for several clinical outcomes, although this comparison does not establish pharmacological synergy. The short treatment duration and the mixed spondylitis/spondylosis population limit the generalizability of the findings [86].

#### 3.5.2. Alzheimer’s Disease

Alzheimer’s disease (AD) represents a neurodegenerative disorder in which progressive neuronal injury is accompanied by sustained neuroinflammatory and immune activation. Clinical evidence for curcumin in established AD remains limited and inconsistent. In the updated meta-analysis by Wang et al. [87], the subgroup of patients with AD did not show a significant pooled benefit, despite an overall beneficial effect on global cognition across the broader study population. This finding indicates that cognitive effects observed in non-demented or mixed populations cannot be extrapolated directly to established neurodegenerative disease.

Negative primary evidence includes the randomized trial of Ringman et al., in which 36 patients with mild-to-moderate Alzheimer’s disease received the placebo or *Curcuma* C3 Complex^®^ at 2 or 4 g/day for 24 weeks. No significant between-group differences were found in clinical or biomarker efficacy measures. These findings do not support the efficacy of this preparation under the conditions studied, although the small sample limits definitive conclusions [67].

Clinical evidence for *Boswellia* is even more limited but includes a randomized double-blind placebo-controlled trial in 85 patients with mild-to-moderate AD. Treatment for six months with K-Vie™, a *Boswellia serrata* extract standardized to 40% boswellic acids, improved scores on the Mini-Mental State Examination (MMSE) and the Clinical Dementia Rating–Sum of Boxes (CDR-SOB) compared with the placebo. Circulating inflammatory cytokines, including IL-6 and TNF-α, were reduced at the one-month assessment [26]. These findings are of interest because they link clinical improvement with modulation of systemic inflammatory markers, but confirmation in independent trials using the same formulation is required.

### 3.6. Acute Systemic Inflammatory Disorders with Immune Dysregulation

#### 3.6.1. COVID-19

For curcumin, much of the clinical evidence in acute systemic inflammatory conditions comes from COVID-19. Sawangjit et al. [69] included 23 randomized trials involving 1407 patients. The interventions included SinaCurcumin, Curcuden, curcuRouge and other turmeric-containing preparations, including multi-ingredient combinations. Table 3 identifies the clinical reports without assigning independent-cohort totals to formulations. The pooled analysis suggested lower all-cause mortality (RR 0.39; 95% CI 0.23–0.67; eight trials), less mechanical ventilation and clinical deterioration, and greater symptom resolution. Nine trials were judged to be at a high risk of bias. Differences in disease severity, standard care, formulation and dose limit interpretation; effects of multi-ingredient combinations cannot be attributed to curcumin alone. These studies do not establish a formulation-specific dose–response relationship. A separate meta-analysis of six randomized trials involving 333 hospitalized patients suggested benefits of nano-curcumin for mortality and selected inflammatory biomarkers [88]. Because the syntheses may include overlapping primary studies, their trial counts must not be added together. Findings remain preliminary and specific to the preparations and clinical settings investigated.

For *Boswellia*, controlled clinical evidence is limited and currently centered on COVID-19. In a randomized double-blind placebo-controlled trial in 47 hospitalized patients with moderate COVID-19, Inflawell^®^, a *Boswellia serrata* extract standardized to 40% boswellic acids, was administered at 999 mg/day in addition to standard care. Treatment shortened hospitalization and improved the neutrophil-to-lymphocyte ratio compared with the placebo [72]. Subsequent analyses showed reductions in circulating TNF-α, IL-1α, IL-8 and MCP-1 and modulation of the TNF-α/NF-κB pathway [73]. Another small randomized trial combining boswellic acids with glycyrrhizin reported a shorter recovery time and fewer deaths than the placebo, but the contribution of *Boswellia* cannot be separated from that of glycyrrhizin [74]. The small sample in the Inflawell trial (*n* = 47) limits the precision and generalizability of the clinical findings; related biomarker analyses do not constitute independent clinical replication.

#### 3.6.2. Sepsis

Evidence for curcumin in sepsis and other critically ill populations remains limited. In a randomized double-blind placebo-controlled trial in 40 critically ill patients, nanomicellar curcumin administered for 10 days reduced expression of NLRP3, NF-κB and IFN-γ and decreased circulating IL-17, IL-22 and HMGB1, accompanied by improvements in Sequential Organ Failure Assessment (SOFA) and multiple organ dysfunction scores [76]. A subsequent randomized double-blind trial in 66 patients evaluated curcuminoids combined with piperine (1000 mg curcuminoids plus 10 mg piperine/day for 7 days) and provided further clinical evidence for curcumin-based adjunctive therapy [75]. A broader meta-analysis of seven randomized trials involving 571 critically ill patients also suggested a clinical benefit, although the included populations were not restricted to sepsis [89]. In particular, the 40-patient trial provides imprecise estimates that require independent confirmation; concurrent improvements in biomarkers and clinical scores do not establish an immune mechanism of clinical benefit.

Reported changes in inflammatory biomarkers should be distinguished from effects on clinical outcomes. Even when both are observed, their concurrence does not establish that modulation of a specific immune pathway caused the clinical benefit. Given the limitations and heterogeneity of the available trials in COVID-19 and sepsis, these findings should be regarded as preliminary and specific to the preparations and clinical settings investigated.

No controlled clinical evidence for *Boswellia* in sepsis was identified; the available evidence remains preclinical.

### 3.7. Safety and Potential Drug Interactions

Safety cannot be inferred from a botanical origin or improved bioavailability. Small, short-term clinical trials provide limited information on uncommon serious adverse reactions and long-term tolerability. Safety findings should therefore be interpreted in relation to the preparation, constituent composition, dose and treatment duration investigated.

Gastrointestinal tolerability should be distinguished from both potential local therapeutic activity and the experimental gut–liver-axis hypothesis discussed below. In a single-dose escalation study in 24 healthy volunteers, adverse events were mild (grade 1), without an apparent dose relationship [90]. This short-term study does not establish chronic gastrointestinal safety or demonstrate intestinal tissue injury. Changes in microbiota composition alone likewise should not be labeled gastrointestinal toxicity without evidence of adverse functional or tissue effects.

Turmeric-associated liver injury has been documented in the US Drug-Induced Liver Injury Network. Among ten adjudicated cases, injury was predominantly hepatocellular; five patients were hospitalized and one died of acute liver failure. Seven patients carried HLA-B*35:01, indicating a strong association with genetic susceptibility [91]. This association is consistent with an immune-mediated component but does not establish a sufficient genetic cause or a validated basis for routine predictive screening. Piperine was detected in three of seven analyzed products. Subsequent reports also describe liver injury following combined turmeric and black pepper consumption [92]. These observations warrant attention but do not establish that piperine or enhanced bioavailability caused the injury; nor do they permit reliable comparisons of risk across delivery technologies.

A subsequent clinicopathological series described eleven patients, with turmeric causality classified as probable in eight and possible in three [93]. A 2026 case report described recurrent, more severe autoimmune-like hepatitis after re-exposure to a turmeric supplement containing black pepper extract [94]. These reports do not establish the incidence or permit a comparative ranking of formulations by hepatotoxicity. Suspected supplement-associated liver injury warrants withdrawal of the implicated preparation and clinical assessment; re-exposure should be avoided.

Formulation-related effects on the gut–liver axis provide a further, experimentally based hypothesis [95]. In mice, carboxymethylcellulose and polysorbate 80 altered the intestinal microbiota and promoted intestinal inflammation and metabolic disturbances [96]. Experiments using human-derived gut microbiota also demonstrated emulsifier-dependent changes in microbial composition and activity in vitro [97]. These findings concern the emulsifiers and experimental conditions tested; they do not demonstrate hepatotoxicity of curcumin formulations or justify generalization to all emulsifiers. A controlled feeding study in healthy volunteers also found changes in the microbiota and metabolome with carboxymethylcellulose at 15 g/day for eleven days [98]; this was not a study of curcumin formulations and did not demonstrate liver injury.

Similarly, intravenously administered poly(lactic-co-glycolic acid) nanoparticles altered gut microbial composition and hepatic gene expression, including pathways related to mitochondrial metabolism, in an obesity mouse model [99]. The administration route, experimental model and absence of a tested oral curcumin formulation substantially limit clinical extrapolation. Whether particular excipients or nanocarriers contribute to human turmeric-associated liver injury through the gut–liver axis remains unresolved.

Drug interactions represent a separate safety concern. In patients receiving tamoxifen, curcumin combined with piperine reduced exposure to the active metabolite endoxifen by a mean of 12.4%; curcumin alone produced a smaller, statistically non-significant reduction [100]. This demonstrates a pharmacokinetic interaction under the conditions studied, although effects on cancer outcomes were not evaluated. Experimental effects on drug-metabolizing enzymes or transporters should not be assumed to predict the direction or magnitude of clinical interactions. In eight healthy subjects, a single 2 g curcumin dose increased exposure to a therapeutic 2 g sulfasalazine dose approximately 3.2-fold, consistent with intestinal BCRP inhibition [101]. This result cannot be extrapolated quantitatively to every formulation or co-medication.

For *Boswellia*, extracts have inhibited several CYP enzymes in vitro, and boswellic acids have modulated *p*-glycoprotein transport activity in experimental assays [102,103]. Spontaneous reports have also described increased anticoagulation during concomitant use of *Boswellia* products and warfarin [104]. These reports constitute a clinical safety signal rather than definitive proof of a formulation-independent interaction. The findings obtained with whole extracts should not automatically be attributed to individual boswellic acids, and clinical interaction data remain limited. KBA and AKBA also modulated OATP1B3 and MRP2 in experimental assays [105]; the magnitude and clinical occurrence of these interactions at oral doses remain uncertain.

Overall, neither enhanced exposure nor the absence of serious adverse events in small trials establishes long-term safety. Future studies should document the formulation composition, excipients, concomitant medication, adverse events and liver biochemical findings sufficiently to support preparation-specific benefit–risk assessment.

## 4. Discussion

Across the selected indications, clinical studies indicate recurring evidence of a benefit for curcumin and, on a smaller evidence base, for *Boswellia* preparations. However, the magnitude and consistency of these effects vary between indications and preparations. The clinically relevant question is therefore not simply whether curcumin or *Boswellia* is effective, but which preparation and exposure produce a reproducible treatment effect in a given clinical setting.

A central finding of this review is that the formulation technology and active-constituent composition complicate dose comparisons across curcumin and *Boswellia* preparations. Similar nominal doses may deliver different constituents and produce different systemic exposures. Dose comparability therefore requires consideration of the composition, formulation, dosing regimen and pharmacokinetic data available for the constituents concerned. Differences in exposure should be distinguished from formal pharmacokinetic bioequivalence, which requires sufficiently similar AUC and C_max_ values under comparable conditions, assessed against predefined acceptance limits.

Clinical dose equivalence remains a separate question. Demonstrating equivalent clinical effects requires appropriately designed comparative trials in a defined indication, with predefined, clinically justified equivalence margins. Separate demonstrations of superiority over the placebo, or the absence of a statistically significant difference between treatments, do not establish equivalence. Formulation-specific dose-finding and independently replicated confirmatory trials are needed to establish effective dosing regimens; direct comparative trials are additionally required to determine whether different preparations provide equivalent clinical effects.

Interpretation is further complicated by overlapping primary studies across systematic reviews, differences in trial selection and discrepancies between doses reported in reviews and those administered in the original studies. These limitations restrict dose-based subgroup analyses, indirect comparisons and network meta-analyses. The available evidence therefore supports a focus on dose comparability rather than an assumption of interchangeable doses or therapeutic superiority based on enhanced bioavailability.

Higher systemic exposure does not by itself establish greater clinical efficacy. Plasma measurements may not adequately reflect exposure at the relevant site of action. In a randomized crossover study of 12 healthy volunteers, standard and phosphatidylcholine curcumin preparations produced different plasma and rectal-tissue exposure profiles; plasma measurements did not provide a consistent positive relationship with mucosal concentrations [106]. These findings support assessing intestinal tissue exposure separately from plasma exposure, but do not establish clinical efficacy. Local gastrointestinal effects may contribute to outcomes in intestinal disorders, whereas distribution to other target tissues remains insufficiently characterized in humans. Tissue distribution, metabolism and differences in active-constituent composition therefore require preparation-specific investigation. Preparations containing predominantly hydrogenated curcuminoids, such as CuroWhite™, additionally differ chemically from native-curcumin preparations and should not be compared solely on the basis of the nominal dose [18].

Cognitive studies provide a further example of the difficulties arising from formulation heterogeneity. In the meta-analysis by Wang et al. [87], preparations classified as having enhanced bioavailability showed a significant pooled effect on global cognition (SMD 1.57; 95% CI 0.29–2.84; *p* = 0.016), whereas the effect was not significant for non-enhanced preparations (SMD 0.31; 95% CI −0.21–0.84; *p* = 0.238). However, heterogeneity within the enhanced-bioavailability subgroup was very high (*I*^2^ = 94%). Moreover, Longvida^®^, a solid-lipid curcumin formulation, was classified as non-enhanced in that analysis. This classification, together with the marked heterogeneity, limits the interpretation of the subgroup comparison as evidence that enhanced bioavailability itself explains the observed difference in cognitive outcomes.

Exercise-related studies provide another example, as trials using pharmacologically distinct preparations, including Cureit™ and Meriva^®^, as well as conventional curcumin preparations, were pooled across different nominal doses and dosing schedules, limiting the interpretation of formulation-independent dose–response relationships [107].

For nutrition-oriented research and the development of evidence-based nutraceuticals, the botanical source, extract composition, formulation technology, relevant constituent content, pharmacokinetic characteristics and administered dose should therefore be reported explicitly. Comparative human pharmacokinetic studies should characterize exposure differences between preparations. Formulation-specific dose-finding and adequately powered, independently replicated clinical trials should establish effective dosing regimens, while clinical equivalence between preparations requires direct comparisons designed to test equivalence.

## 5. Conclusions

*Curcuma* and *Boswellia* preparations show potential for an adjunctive symptomatic benefit in selected inflammatory disorders, with comparatively more clinical evidence in knee osteoarthritis. Findings in other indications remain inconsistent or limited, particularly in Alzheimer’s disease, COVID-19 and sepsis. Experimental pathway effects and changes in human inflammatory biomarkers should not be equated with a demonstrated beneficial modulation of immune function.

The preparation composition, formulation technology, dose and treatment duration limit comparisons across trials. Increased bioavailability alone does not establish greater clinical efficacy, and clinical dose equivalence between preparations remains unproven. Safety assessment must also consider idiosyncratic liver injury and potential drug interactions, without generalizing incompletely characterized reports to all formulations. Interpretation should account for methodological limitations, overlapping study populations and reported industry relationships. Future research should combine formulation-specific human pharmacokinetics and dose-finding with adequately powered, independently replicated clinical trials using patient-relevant outcomes.

## Figures and Tables

**Table 3:** Selected clinical evidence by disease and preparation.

Clinical Setting	Preparations Investigated	References by Preparation
Rheumatoid arthritis	*Curcuma*: other oral preparations, Cureit, CuroWhite, nanomicellar curcumin and BCM-95. CuroWhite contains hydrogenated curcuminoids.	Other oral curcumin: [27]; Cureit™: [28]; CuroWhite™: [18]; Nanomicellar curcumin: [27,29,30]; Rezaieyazdi 2023 in [27]; BCM-95: [31]
Ulcerative colitis	Oral curcumin preparations, including nanomicellar curcumin. *Boswellia*: Casperome (observational evidence).	Other oral curcumin: [32] (Mohseni, Table 1), including Banerjee 2017/2021 [33,34]; Nanomicellar curcumin: [32,35]; Casperome: [21] (observational)
Crohn disease	*Curcuma*: other oral preparations and Theracurmin. *Boswellia*: H15 and Boswelan.	Other oral curcumin: [32,36]; Theracurmin^®^: [32,37]; H15: [38]; Boswelan: [39]
Knee osteoarthritis	*Curcuma*: other turmeric preparations, nanomicellar curcumin, Theracurmin, Longvida, BCM-95/CuraMed, Curcugen, Meriva, Curene and CurQfen. *Boswellia*: other extracts, Aflapin, 5-Loxin, Boswellin and Boswellin Super. Combinations: Curamin, LI73014F2 and CLBS.	Other oral curcumin: [40,41,42,43,44,45,46,47]; Nanomicellar curcumin: [48,49]; Theracurmin^®^: [50]; Longvida^®^: [51]; BCM-95/CuraMed: [52,53,54,55]; Curcugen^®^: [56]; Meriva^®^: [57] (nonrandomized); Curene^®^: [58]; CurQfen^®^: [59]; Other *Boswellia* extracts: [60]; Aflapin^®^/AprèsFlex^®^: [22,23,61,62]; 5-Loxin^®^: [22,25]; Boswellin: [63]; Boswellin Super: [64]; Curamin (curcumin + *Boswellia*): [52]; LI73014F2: [65]; *Curcuma* + *Boswellia* blend (CLBS): [66]
Alzheimer’s disease	*Curcuma*: conventional preparations. *Boswellia*: K-Vie.	Other oral curcumin: [67,68]; K-Vie™: [26]
COVID-19	*Curcuma*: SinaCurcumin, Curcuden, curcuRouge and other turmeric-containing interventions, including combinations. *Boswellia*: Inflawell; boswellic acids with glycyrrhizin.	Other turmeric-containing interventions: (Sawangjit et al. [69], Table 2 in that publication); SinaCurcumin^®^: (Sawangjit et al. [69], Table 2 in that publication); Curcuden: [69,70]; curcuRouge^®^: [69,71]; Inflawell^®^: [72,73]; Boswellic acids + glycyrrhizin: [74]
Sepsis	Nanomicellar curcumin and curcuminoids with piperine. No controlled clinical *Boswellia* evidence identified in the selected sources.	Curcuminoids with piperine: [75]; Nanomicellar curcumin: [76]

Reading guide: Each row represents one clinical setting. The middle column identifies the preparations; the right column links each preparation to its sources. Numbers in square brackets are references, not trial counts. This is a selected narrative overview, not an exhaustive inventory or a grading of efficacy or evidence certainty. Entries refer to randomized trials unless labeled observational or nonrandomized. Multi-ingredient combinations do not establish effects of an individual botanical. Haroyan et al. (2018) investigated CuraMed and Curamin within the same trial; this publication therefore supports both preparation entries [52]. Similarly, Sengupta et al. (2010) compared Aflapin and 5-Loxin with placebo within a single trial [22]. Shep et al. (2019 and 2020) reported the same trial registration number (ISRCTN10074826) and the publications are therefore not treated as independent trials unless separate recruitment can be established [53,54]. Banerjee et al. (2017 and 2021) appear to represent a conference report and a subsequent full publication from the same study period [33,34]. Mohseni et al. list these reports separately in Table 1 [32]. However, their reported sample sizes differ, so the extent of participant overlap remains uncertain. The oral-curcumin UC selection excludes the *Curcuma*–QingDai combination and rectal enema study. COVID-19 preparation assignments follow Table 2 in the systematic review by Sawangjit et al. [69]. Evidence from broader critically ill populations is not automatically classified as sepsis evidence. CLBS, *Curcuma*–*Boswellia* blend; LI73014F2, *Terminalia chebula*–*Curcuma longa*–*Boswellia serrata* combination. Other preparations are heterogeneous and must not be treated as pharmacologically interchangeable.

## Data Availability

No new data were created or analyzed in this study. Data sharing is not applicable to this article.

## Abbreviations

The following abbreviations are used in this manuscript:

AKBA	acetyl-11-keto-β-boswellic acid
KBA	11-keto-β-boswellic acid
PK	pharmacokinetics
